# Influence of the Agricultural Conservation Easement Program wetland practices on winter occupancy of Passerellidae sparrows and avian species richness

**DOI:** 10.1371/journal.pone.0210878

**Published:** 2019-01-24

**Authors:** Katharine E. Lewis, Christopher T. Rota, Christopher M. Lituma, James T. Anderson

**Affiliations:** Davis College of Agriculture, Natural Resources, and Design, West Virginia University, Morgantown, West Virginia, United States of America; Seoul National University, REPUBLIC OF KOREA

## Abstract

Wetlands enrolled in the Agricultural Conservation Easement Program (ACEP) are established as a means of restoring wetland ecosystems and wildlife habitat on private, agricultural land. In West Virginia, USA, ACEP wetlands have never been evaluated to determine how they function as wildlife habitat in comparison to other available wetland habitat in the state. We measured the wintering occupancy of Passerellidae species and apparent avian species richness on ACEP wetlands and a set of reference wetlands located on public land in West Virginia to evaluate if ACEP wetlands are being used similarly by avian species to other available wetland habitat in the state. Apparent avian species richness and the occupancy probability of four Passerellidae species—song sparrows (*Melospiza melodia*), dark-eyed juncos (*Junco hyemalis*), swamp sparrows (*Melospiza georgiana*), and white-throated sparrows (*Zonotrichia albicollis*)—did not differ between ACEP and reference sites. In addition to other vegetative and habitat associations for each species, dark-eyed junco occupancy was negatively correlated with wetland size while swamp sparrow occupancy and apparent avian species richness were positively associated with wetland size. These results indicate that ACEP wetlands are providing winter avian habitat as well as another source of wetland habitat in the state. Maintaining and expanding ACEP wetlands in West Virginia would continue to provide wetland systems in areas that are otherwise lacking these habitats.

## Introduction

In North America, >50% of wetlands have been lost to drainage, urban development and agricultural development over the last 200 years [1]. Remaining wetlands are often exposed to agricultural runoff and physical impacts from livestock grazing [2]. Because wetlands provide important ecosystem services [3–6], including providing wildlife habitat [7], many wetland conservation programs and policies in the U.S. are aimed at reversing past losses [7]. One of these initiatives, the Agricultural Conservation Easement Program (ACEP; formerly the Wetland Reserve Program) is a platform administered through the United States Department of Agriculture’s Natural Resources Conservation Service (NRCS). This program provides financial and technical assistance to landowners in restoring wetlands on their property across the United States by purchasing and administering conservation easements. The objectives of ACEP wetland easements are to provide wetland ecosystem services in agricultural areas, including the creation of wildlife habitat [8]. Since its inception in 1996, over 500,000 ha of wetlands have been restored or created through this program [9]. Between 1996 and 2012, 24 conservation easements were enrolled in the Wetland Reserve Program and ACEP in West Virginia, USA, totaling approximately 179 ha of wetlands.

With any conservation program, it is important to monitor the associated management practices to determine if objectives are being met. Within West Virginia, several studies have aimed at evaluating objectives of wetland management projects. These studies compared vegetative and aquatic macroinvertebrate communities and wildlife use between wetlands created as part of a mitigation process (i.e., creating wetlands in one area to offset losses elsewhere) and reference wetlands. These studies found that mitigated wetlands had similar vegetative cover to naturally occurring sites, although they were composed of younger successional species [10]. Mitigated wetlands also had similar aquatic macroinvertebrate density and diversity [11] and had similar or higher avian and anuran species richness, abundance, and diversity relative to naturally occurring reference wetlands [12]. Another study compared wintering waterfowl use of two created wetlands in West Virginia of differing ages, finding that the newer wetland had comparable waterfowl communities to the older site [13]. With the exception of the winter waterfowl study, all of the above studies occurred during summer months.

While these studies provided valuable insight into the difference between mitigated and naturally occurring wetlands, mitigated wetlands differ from conservation easements in important ways. In particular, mitigation wetlands are typically created or restored to compensate for the loss of other wetland habitat due to drainage or development [7]. Wetlands enrolled in ACEP are located on private, agricultural land in areas that historically had wetland hydrology and vegetative communities. There is, therefore, much to be learned about how well ACEP wetlands meet program objectives within West Virginia, particularly outside the relatively short summer breeding season.

Within West Virginia, the wetlands enrolled in ACEP are classified as freshwater emergent, scrub-shrub, or forested wetlands [8]. The vegetation associated with these wetland types provide resources for passerine species throughout the year. Emergent sedges and other grasses present on freshwater-emergent wetlands provide avian nesting habitat and screening cover from predation [14,15]. The vegetative structural diversity present on scrub-shrub or forested wetlands also influences passerine wetland use and promotes species richness by providing differing vegetation heights and types that align with a broad range of species’ nesting and roosting habitat requirements [16]. A small number of snags and deciduous trees can contribute to the vertical and horizontal vegetative diversity of a wetland that may also influence overall species richness by creating diverse habitat conditions that fulfill different species’ habitat requirements [15].

New world sparrows in the family Passerellidae are a group of birds that associate with vegetative characteristics typical of emergent, forested, and scrub-shrub wetlands. For example, swamp sparrow (*Melospiza georgiana*) abundance is positively associated with vegetative complexity in early successional scrub-shrub habitats, and species such as song sparrows (*Melospiza melodia*) and white-throated sparrows (*Zonotrichia albicollis*) are abundant in these types of habitat [17]. Dark-eyed juncos (*Junco hyemalis*) use tree stands and fields with shrub or tree buffers for foraging and roosting [18]. Within West Virginia, many Passerellidae sparrows such as dark-eyed juncos and white-throated sparrows occur primarily during the non-breeding winter months. In the winter, wetland vegetation provides foraging habitat, thermal cover, and protection from predation in the form of dense shrubs, herbaceous material, and emergent vegetation [19,20]. Species such as song sparrows and white-throated sparrows select winter foraging plots with dense screening cover or forage in areas closer to screening cover to reduce the risk of predation [19,21]. Additionally, winter site selection of Passerellidae sparrows may be impacted by broader landscape-scale factors in addition to site-level vegetative characteristics. For example, swamp sparrows’ occupancy and abundance are positively associated with wetland size [15,22]. In addition to having associations with vegetation typically found in wetlands, many Passerellidae sparrows are frequently found within early successional agricultural landscapes, and have been previously used to evaluate early successional habitat [17]. Passerellidae sparrows are therefore a well-suited group of birds for evaluating the ability of ACEP wetlands to provide winter wildlife habitat in West Virginia.

In addition to evaluating habitat use of particular species associated with wetland vegetation, insight into the ability of ACEP wetlands to provide winter wildlife habitat can be obtained by evaluating avian species richness between ACEP and reference wetlands. Because ACEP wetlands were located within an agricultural landscape, they were adjacent to different types of habitat such as open fields and shrub or tree lines. Avian species richness overall and across different habitat guilds (i.e., generalist, forest or shrub specialists) is positively associated with habitat heterogeneity [23], so the presence of habitats associated with agricultural working lands may increase species richness. However, within agricultural landscapes, species richness can vary across an intensity gradient. For example, species richness of forest specialist birds was greatest in low intensity agriculture areas, while generalist species were dominant in high intensity pasture [24]. Consequently, increases in species richness associated with increased heterogeneity could be offset by species losses in certain agricultural settings such as high intensity pasture or row crops. Comparing species richness between ACEP and reference wetlands could therefore give insight into the ability of ACEP wetlands to function as wildlife habitat for a diverse set of avian species.

For this study, we evaluated the ability of ACEP conservation easements to provide wildlife habitat by comparing (1) occupancy probability of wintering Passerellidae species and (2) avian species richness between ACEP wetlands on private agricultural wetlands and reference wetlands located on public land, while statistically controlling for vegetative characteristics which could influence occupancy. We hypothesized that: (1) occupancy probability of Passerellidae sparrows would be equal to or higher on ACEP wetlands relative to reference wetlands due to the habitat heterogeneity provided by the agricultural matrix surrounding ACEP wetlands; and (2) apparent avian species richness would also be equal or higher on ACEP wetlands relative to reference wetlands owing to the immediate landscape around ACEP wetlands and their overall vegetative similarities to reference wetlands. Agriculture has been a leading cause of wetland losses in the United States [25] and worldwide, but conservation of wetlands within agricultural landscapes can also be an important source of wetland habitats [1]. Conservation practices such as ACEP provide a means of conserving wetland habitat while enabling continued human use of the land. Our study aims to evaluate avian use of ACEP sites to determine if set-aside conservation areas within agricultural landscapes are effective at providing wildlife habitat.

## Materials and methods

### Study area

**Fig 1:**
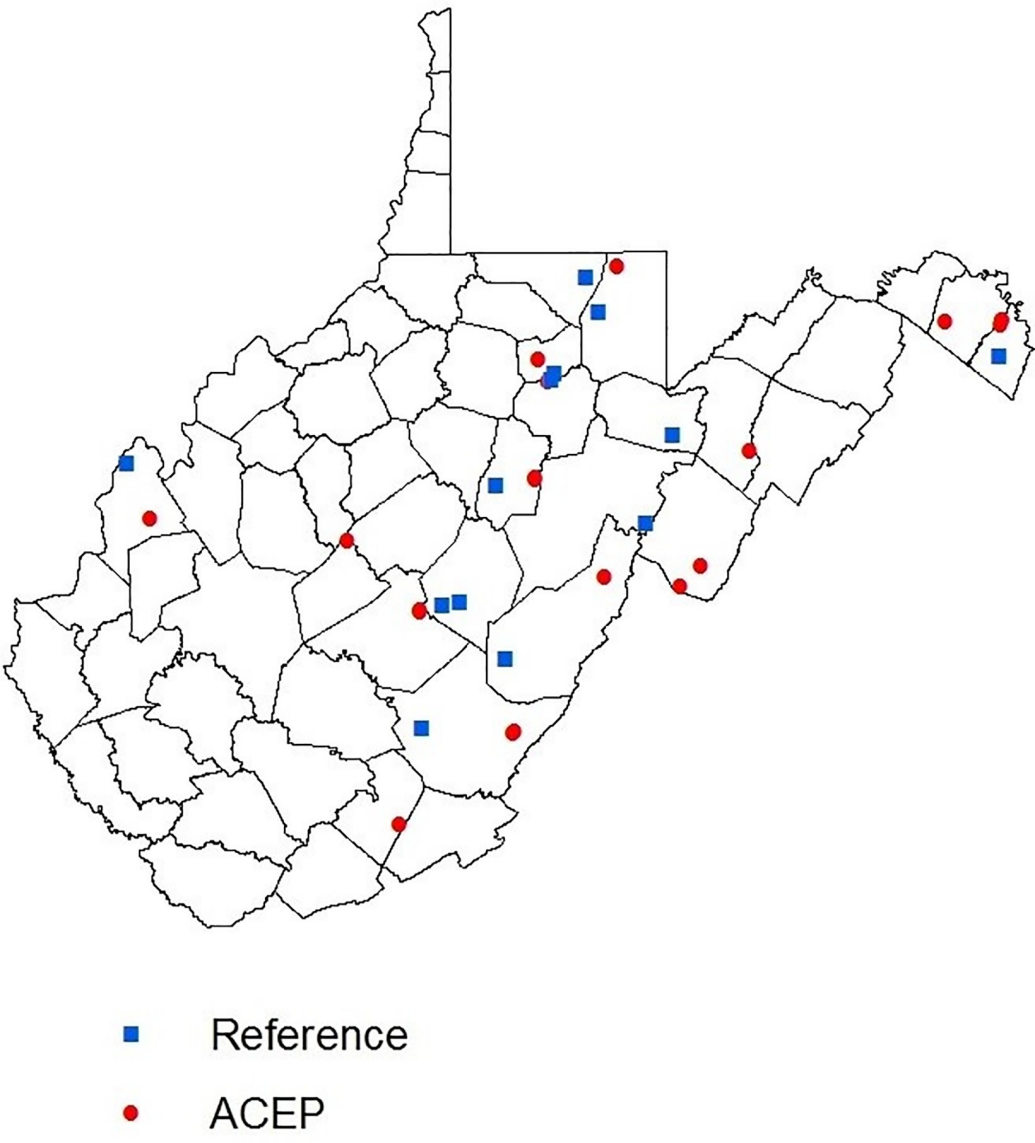
Locations of Agricultural Conservation Easement Program (ACEP) and reference wetlands in West Virginia, USA, in the winters of 2016–2017 and 2017–2018. ACEP properties were administered through the Natural Resources Conservation Service in West Virginia, USA. Reference wetlands were located on public land on West Virginia Division of Natural Resources Wildlife Management Areas and State Parks, and The Nature Conservancy land.

Our study occurred within NRCS ACEP wetlands located primarily on private lands throughout West Virginia, USA, and on nearby wetlands located on public land (Fig 1). We included all West Virginia wetlands enrolled in ACEP with the exception of four sites where we were denied access by the landowners. ACEP wetland sites were located on private land and were not open to the public with the exception of one site managed by the West Virginia Division of Natural Resources (WVDNR). The ACEP sites were located within an agricultural landscape predominately composed of pasture, with one site being surrounded by actively cropped corn fields (*Zea mays*). Wetlands located adjacent to active livestock pasture had fencing to exclude livestock. The site managed by the WVDNR was actively managed for water depth; however, all other ACEP sites were not actively managed to promote naturally sustained habitat with little human intervention. All ACEP sites were classified as either freshwater palustrine emergent, forested, or scrub-shrub wetlands [26]. As specified by the Cowardin classification system [26], not all sites had continuous, standing open water. Some sites were composed of hydric soils that allowed for hydrophytic vegetation and flooding during some portion of the year. Emergent wetlands were dominated by rooted hydrophytic vegetation such as cattails (*Typha* spp.), bulrush (*Scirpus* spp.), and sedges (*Carex* spp.). Forested wetlands had an overstory of trees such as black willow (*Salix nigra*) and American sycamore (*Platanus occidentalis*) and were dominated by woody vegetation >6 m tall. Scrub-shrub wetlands were dominated by woody vegetation <6 m tall such as alders (*Alnus* spp.) and buttonbush (*Cephalanthus occidentalis*). ACEP wetlands ranged in size from <0.4 ha to 32 ha, and the average size of all of the ACEP wetlands included in this study was 8.69 ha (S1 Table). We determined area of the ACEP wetlands from a shapefile containing polygons of the boundaries of each wetland easement delineated by the NRCS in the state in ArcMap 10.4 [27].

Reference wetlands were located on public land in Wildlife Management Areas (WMAs) or State Parks owned by the WVDNR, or property owned by The Nature Conservancy. Some reference wetlands were open for public access and use such as hunting, but reference wetland sites were not actively managed. We selected reference wetlands to be as similar to ACEP wetlands as possible. To do this, we used the National Wetlands Inventory data layer [27] in ArcMap 10.4 [28] to first identify wetlands in WMAs, State Parks, or property owned by The Nature Conservancy. The National Wetlands Inventory data layer contained polygons of all wetland habitats in West Virginia, and each polygon contained information about the wetland area and type according to the Cowardin classification system [26]. We then limited this list to include only wetlands that were ≤ 32 ha; classified as emergent, scrub-shrub, or forested; and in the same or an adjacent county as ACEP wetlands. The average reference wetland size was 8.87 ha and ranged in size from <0.4ha—25.7 ha. Because of their location within State Forests, WMAs and The Nature Conservancy land, reference wetlands were often adjacent to forested areas. Wetland habitat in West Virginia is scarce: approximately 80% of West Virginia is forested [29], while <1% of the state’s surface is covered by wetlands [30]. As a consequence, there were fewer reference sites than ACEP sites that fit all of these criteria, so we included all potential reference sites in the study.

### Point counts

**Fig 2:**
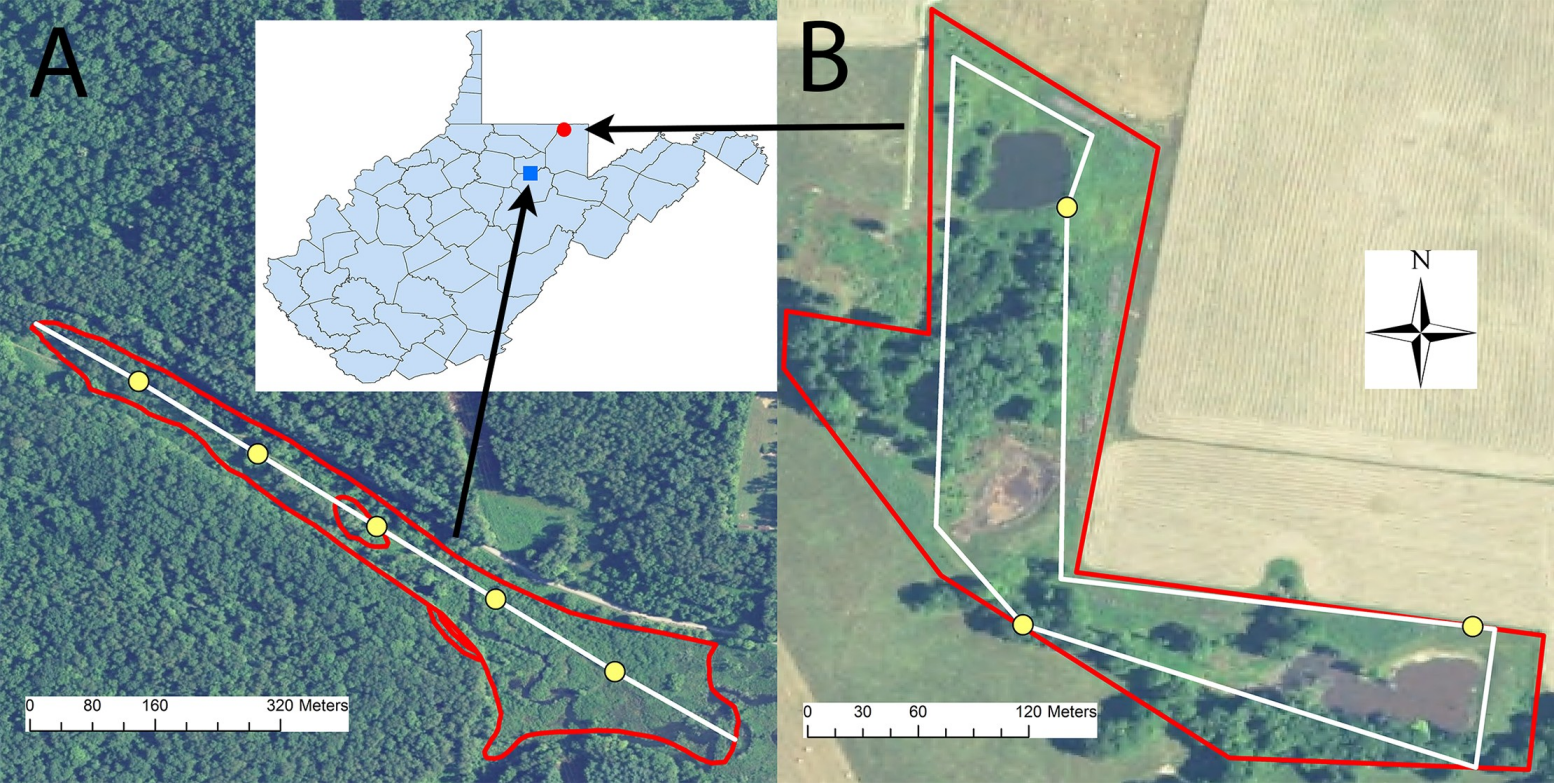
Placement of point count surveys to determine sparrow occupancy and species richness at wetlands in West Virginia, USA in the winters of 2016–2017 and 2017–2018. Figure A represents placement of point counts along transect line through the centroid of a reference wetland. Figure B represents placement of point counts along transect placed 50 m from the wetland edge in an ACEP wetland. Yellow dots represent point count locations. White lines represent transects along which point counts were placed. Red polygons represent wetland boundaries.

To determine Passerellidae occupancy and all species richness, we conducted point counts throughout each of the sites during the winters of 2016–2017 and 2017–2018 from 1 November to 15 February each year. At all sites we first created transects within wetland areas, and transect delineation varied depending on the characteristics of the wetland site. For sites that had permanent standing water, we placed transects along a buffer placed at least 50 m from the water’s edge. We then placed a transect to roughly follow the contours of the wetland boundary, allowing for adjustments for field conditions that made some areas impassible such as areas of dense shrubs or multiflora rose (*Rosa multiflora*). For wetlands with no standing water (e.g., small streams or saturated soils), we placed a transect through the centroid of wetland sites running lengthwise across the site (Fig 2). We then placed point counts along these transects. We randomly selected the location of the first point count along each transect and placed point counts 150 m apart along the rest of the transect. After plotting the points, we manually checked the distance between points that were across from one another to ensure that those points were at least 150 m apart. For those that were less than 150 m apart, we removed the fewest number of points that would result in all remaining points being > = 150 m apart. Because the size and shape of each wetland varied, the length of the transect and the number of point counts varied by wetland (S1 Table).

We recorded all species seen and heard during unlimited radius, single observer, 10-minute point counts. We conducted our point counts starting a half hour before sunrise and concluded by 1500 each day. We used visual and auditory detection to determine species presence and recorded the approximate distance to the individual bird using distance categories of <50 m, 50–100 m, and >100 m [31]. We employed removal sampling during the point counts, meaning that we only recorded an individual bird the first time it was detected, even if it continued to be detected throughout the 10-minute survey. Each wetland site was visited twice each year by the same observer between November and February 2016–2017 and 2017–2018.

### Detection probability covariates

During point counts, we recorded several variables thought to influence detectability. We classified sky condition as clear skies, partly cloudy, overcast, light precipitation, and heavy precipitation. Point counts were not conducted in sky conditions categorized as heavy precipitation. We measured noise disturbance level with a sound level meter that recorded noise to the nearest 0.5 decibel. We measured wind speed in meters per second with a Kestrel 1000 hand-held anemometer, and we recorded temperature in degrees Celsius. We also recorded time of day and day of year that the point counts were conducted (Table 1).

**Table 1:** Detection and occupancy covariates used to model occupancy of Passerellidae sparrows on Agricultural Conservation Easement Program (ACEP) wetlands and reference wetlands located in West Virginia, USA in the winters of 2016–2017 and 2017–2018.

Covariate Name	Occupancy / Detection	Levels*	Mean (Range)**
Noise disturbance	Detection	Continuous (decibels)	38.8 (0–67)
Wind speed	Detection	Continuous (meters/second)	0.60 (0–4.5)
Sky condition	Detection	Categorical:	
		0–1: clear skies to partly cloudy	0.56
		2–3: overcast to light precipitation	0.44
Temperature	Detection	Continuous (°C)	2.23 (-12.2–17.8)
Time of day	Detection	Continuous (0–24 hours)	10.6 (6.85–15.22)
Day of year	Detection	Continuous (1 = survey day 1, max = last survey day)	36 (1–102)
Herbaceous material	Occupancy	Categorical:	63.7%
		0%—25%	0.22
		26%—75%	0.26
		76%—100%	0.52
Shrub at the 1x1 m scale	Occupancy	Categorical:	13.7%
		0–50%	0.77
		51%—100%	0.23
Bare-ground	Occupancy	Categorical:	21.8%
		0%—50%	0.73
		51%—100%	0.27
Shrub at the 5x5 m scale	Occupancy	Categorical:	38.8%
		0%—25%	0.41
		26%—75%	0.37
		76%—100%	0.22
Water	Occupancy	Categorical:	6.79%
		Absent	0.85
		Present	0.15
Woody Vegetation	Occupancy	Categorical:	2.61%
		Absent	0.87
		Present	0.13
Wetland size	Occupancy	Continuous (ha)	10.8 (0.28–32.4)
Wetland type	Occupancy	Categorical:	NA
		ACEP	0.60
		Reference	0.40
Year	Occupancy	Categorical:	NA
		Year 1	
		Year 2	

*Levels indicate the categories (for categorical covariates) or units (for continuous covariates) the covariate was measured in.

**For categorical variables, we report the proportion of observations that fell within each category

### Occupancy covariates

**Fig 3:**
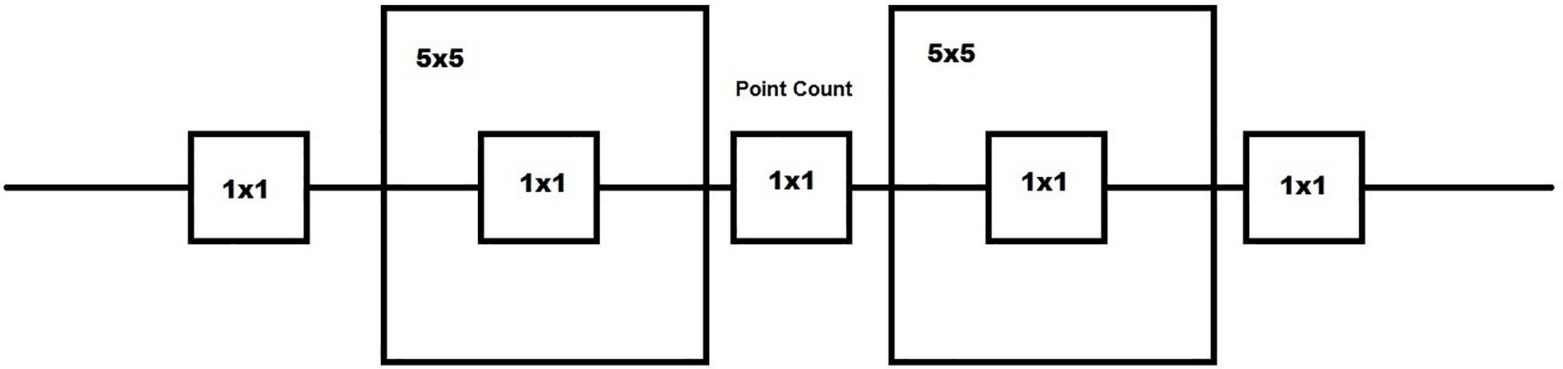
Nested quadrat design used to measure horizontal vegetative cover at each point location on Agricultural Conservation Easement Program (ACEP) and reference wetlands located in West Virginia, USA in the winters of 2016–2017 and 2017–2018. Five 1x1 m quadrats were placed five m apart with the point count location serving as the center 1x1 m quadrat. Within the 1x1 m quadrats, percent cover of herbaceous material, shrub, bare-ground, woody vegetation, and water were measured. Within the 5x5 m quadrats, percent shrub was measured.

We measured vegetative characteristics thought to influence the probability of sparrow occupancy at each point count survey. We measured horizontal vegetative cover using a nested quadrat design [32] (Fig 3). With the point count at the center, we placed five 1x1 m quadrats every 5 m along the transect line to measure horizontal vegetative cover. Within the 1x1 m quadrats, we measured vegetative cover using Daubenmire cover classes [33]. We recorded Daubenmire cover class of herbaceous, scrub-shrub, bare ground, open water, and woody vegetation. The herbaceous category included all forbs and grass species such as goldenrod (*Solidago* spp.), sedge species (*Cyperaceae*), bulrush (*Scirpus* spp.), cattails (*Typha* spp.), and common reed (*Phragmites australis*). The scrub-shrub category was defined as non-herbaceous vegetation that was not a tree (< 6 m tall). Included in this category was multiflora rose, greenbrier (*Smilax* spp.) and button bush (*Cephalanthus occidentalis*). Bare ground was defined as areas that did not have any vegetation including roadways, railroad tracks, unvegetated mud flats and cleared trails. Woody vegetation included trees, snags, logs and fallen branches, and the open water category represented areas that had standing water that was deep enough to obscure vegetation such as in the form of a pond, stream, or flooded areas. Additionally, we placed two 5x5 m quadrats 5 m from the central point count location to measure percent shrub cover at a larger scale, which we also assessed using Daubenmire cover classes [33]. (Table 1). We also tested for co-linearity between each variable and found that variables were not correlated, with the absolute value of the correlation coefficient between all variables <0.5 [34].

### Statistical analyses

#### Single-Species occupancy modeling

We estimated the probability each point count was occupied by each of four Passerellidae species: song sparrows, dark-eyed juncos, swamp sparrows, and white-throated sparrows with single-season Bayesian occupancy models [35]. We included these species because they had the highest number of detections across all surveys. There were < 30 total detections of all other Passerellidae sparrows, so detections of remaining species were too sparse to include in analyses. We only included detections within zero-50 m of the observer to avoid double counting detections.

We fit two models for each species, each of which contained a detection-level and an occupancy-level sub-model. The detection sub-model included covariates that could influence an observer’s probability of detecting an individual bird, conditional on a bird being present at the site. The occupancy sub-model included covariates that could influence the probability that a bird occupied a site [36]. The first model we fit for each species was a global model and included all covariates (listed in Table 1) we hypothesized, *a priori*, would influence detection and occupancy probabilities. In an attempt to create a more parsimonious model, we fit a second reduced model excluding all covariates from the global model whose 50% credible intervals overlapped 0. We compared models with Watanabe-Akaike Information Criterion (WAIC) [37], a Bayesian analog of Akaike’s Information Criterion [38] that is used in model selection. We report results of the most supported model (i.e. the model with the lowest WAIC). We assumed logistic (0, 1) prior distributions on all model coefficients [39]. We fit models with JAGS version 4.3.0 [40] via the jagsUI interface [41] within program R version 3.3.1 [42]. We used four Markov chains to simulate posterior distributions of covariates. We ran each chain for 10,000 iterations and discarded 2,500 iterations as burn-in for the white-throated sparrow and dark-eyed junco models and 5,000 iterations as burn-in for the song sparrow and swamp sparrow models to achieve model convergence. We kept every fifth sample to minimize correlations between draws. For all posterior distributions, Ȓ≈ 1.00, indicating successful model convergence.

#### Species richness

We measured apparent avian species richness as the total number of all species detected at each wetland site (i.e., we treated the wetland, not the point count, as the unit of replication) using the glm function in program R version 3.3.3 [42]. We assumed species richness at each wetland was a Poisson random variable, which we modeled as a function of ACEP or reference wetland type, wetland size, and year.

## Results

We surveyed 197 total points on 20 ACEP and 13 reference sites over both survey years, with 118 point counts on ACEP sites ($\bar{x}$ = 4 surveys per site, se = 0.51, min = 1, max = 9) and 79 point counts on reference sites ($\bar{x}$ = 4 surveys per site, se = 0.50, min = 1, max = 7). Over the two survey winters, we detected 61 avian species overall, 10 of which were Passerellidae sparrows. Over both survey years and between ACEP and reference sites, we detected song sparrows most often out of the Passerellidae species (n = 547 detections), followed by white-throated sparrows (n = 166), dark-eyed juncos (n = 134), and swamp sparrows (n = 68). All Passerellidae species were detected on both ACEP and reference sites except for American tree sparrow (*Spizella arborea*), fox sparrow (*Passerella iliaca*), Savannah sparrow (*Passerculus sandwichensis*), and white-crowned sparrow (*Zonotrichia leucophrys*), which were detected only at ACEP sites (Table 2). We report results only from models with the lowest WAIC score (Tables 3–6).

**Table 2:** Passerellidae species detected on Agricultural Conservation Easement Program (ACEP) and reference wetland sites located in West Virginia, USA in the winters of 2016–2017 and 2017–2018.

Species	Detected on ACEP	Detected on Reference	Number of Individuals
Song sparrow	Yes	Yes	547
White-throated sparrow	Yes	Yes	166
Dark-eyed junco	Yes	Yes	134
Swamp sparrow	Yes	Yes	68
Eastern towhee	Yes	Yes	29
Field sparrow	Yes	Yes	5
White-crowned sparrow	Yes	No	5
American tree sparrow	Yes	No	4
Fox sparrow	Yes	No	2
Savannah sparrow	Yes	No	1

Obtained from detection / non-detection survey data collected in the winters of 2016–2017 and 2017–2018 over both survey years and total number of individuals.

**Table 3:** Model results from song sparrow occupancy analyses obtained from point count surveys on West Virginia Agricultural Conservation Easement Program wetlands (ACEP) and reference sites located in West Virginia, USA in the winters of 2016–2017 and 2017–2018.

Model	Detection Covariates	Occupancy Covariates	K	WAIC	ΔWAIC
**Reduced Model**	Time of day	Wetland type	13	333.9	0.00
	Sky condition	Wetland size			
	Wind speed	**Shrub at the 1x1 m scale**			
	Temperature	Shrub at the 5x5 m scale			
	Noise disturbance				
	**Day of year**				
**Global Model**	Time of day	Herbaceous material	19	342.8	8.90
	Sky condition	Shrub at the 1x1 m scale			
	Wind speed	Bare-ground			
	Temperature	Shrub at the 5x5 m scale			
	Noise disturbance	Water			
	Day of year	Woody vegetation			
		Wetland size			
		Wetland type			
		Year			

Covariates included in reduced models excluding all detection and occupancy covariates whose 50% credible intervals overlapped 0, number of parameters (K) reduced model WAICs and global WAICs, and delta WAIC. Global models included all covariates listed in Table 1 and were the same for each species. Covariates in bold had 95% credible intervals in the reduced model that did not overlap zero.

**Table 4:** Model results from dark-eyed junco occupancy analyses obtained from point count surveys on West Virginia Agricultural Conservation Easement Program wetlands (ACEP) and reference sites located in West Virginia, USA in the winters of 2016–2017 and 2017–2018.

Model	Detection Covariates	Occupancy Covariates	K	WAIC	ΔWAIC
**Reduced Model**	**Time of day**	**Wetland size**	10	102.3	0.00
	Sky condition	Shrub at the 5x5 m scale			
	Wind speed				
	Temperature				
	Noise disturbance				
**Global Model**	Time of day	Herbaceous material	19	116.2	13.9
	Sky condition	Shrub at the 1x1 m scale			
	Wind speed	Bare-ground			
	Temperature	Shrub at the 5x5 m scale			
	Noise disturbance	Water			
	Day of year	Woody vegetation			
		Wetland size			
		Wetland type			
		Year			

Covariates included in reduced models excluding all detection and occupancy covariates whose 50% credible intervals overlapped 0, number of parameters (K) reduced model WAICs and global WAICs, and delta WAIC. Global models included all covariates listed in Table 1 and were the same for each species. Covariates in bold had 95% credible intervals in the reduced model that did not overlap zero.

**Table 5:** Model results from swamp sparrow occupancy analyses obtained from point count surveys on West Virginia Agricultural Conservation Easement Program wetlands (ACEP) and reference sites located in West Virginia, USA in the winters of 2016–2017 and 2017–2018.

Model	Detection Covariates	Occupancy Covariates	K	WAIC	ΔWAIC
**Reduced Model**	Time of day	Year	11	106.9	0.00
	**Wind speed**	**Wetland size**			
	Noise disturbance	Herbaceous material			
	Day of year	Shrub at the 1x1 m scale			
		Water			
**Global Model**	Time of day	Herbaceous material	19	114.8	7.90
	Sky condition	Shrub at the 1x1 m scale			
	Wind speed	Bare-ground			
	Temperature	Shrub at the 5x5 m scale			
	Noise disturbance	Water			
	Day of year	Woody vegetation			
		Wetland size			
		Wetland type			
		Year			

Covariates included in reduced models excluding all detection and occupancy covariates whose 50% credible intervals overlapped 0, number of parameters (K) reduced model WAICs and global WAICs, and delta WAIC. Global models included all covariates listed in Table 1 and were the same for each species. Covariates in bold had 95% credible intervals in the reduced model that did not overlap zero.

**Table 6:** Model results from white-throated sparrow occupancy analyses obtained from point count surveys on West Virginia Agricultural Conservation Easement Program wetlands (ACEP) and reference sites located in West Virginia, USA in the winters of 2016–2017 and 2017–2018.

Model	Detection Covariates	Occupancy Covariates	K	WAIC	ΔWAIC
**Reduced Model**	Noise disturbance	Wetland size	9	121.3	0.00
	Day of year	Herbaceous material			
		**Water**			
**Global Model**	Time of day	Herbaceous material	19	139.4	18.1
	Sky	Shrub at the 1x1 m scale			
	Wind	Bare-ground			
	Temperature	Shrub at the 5x5 m scale			
	Noise disturbance	Water			
	Day of year	Woody vegetation			
		Wetland size			
		Wetland type			
		Year			

Covariates included in reduced models excluding all detection and occupancy covariates whose 50% credible intervals overlapped 0, number of parameters (K) reduced model WAICs and global WAICs, and delta WAIC. Global models included all covariates listed in Table 1 and were the same for each species. Covariates in bold had 95% credible intervals in the reduced model that did not overlap zero.

After controlling for vegetative characteristics, we found no differences in occupancy probability for any species between ACEP and reference sites. Although song sparrows were estimated to occupy a greater proportion of ACEP wetlands relative to reference wetlands when all other variables were held at their mean (ACEP occupancy probability = 0.97, 95% credible intervals (CI) = [0.86, 0.99]; reference occupancy probability = 0.93, 95% CI = [0.69, 0.99]), 95% credible intervals of the wetland type slope coefficient overlapped 0 (log odds ratio = 1.1, 95% CI = -0.9–3.2), indicating no strong effect of wetland type. For all other species, the top models did not include wetland type as a variable (Tables 4–6), meaning estimated occupancy probabilities were equivalent between ACEP and reference wetlands (occupancy probabilities when all variables held at mean: dark-eyed junco = 0.19, 95% CI = [0.02, 0.80]; swamp sparrow = 0.11, 95% CI = [0.001, 0.61]; white-throated sparrow = 0.37, 95% CI = [0.10, 0.86])

**Fig 4:**
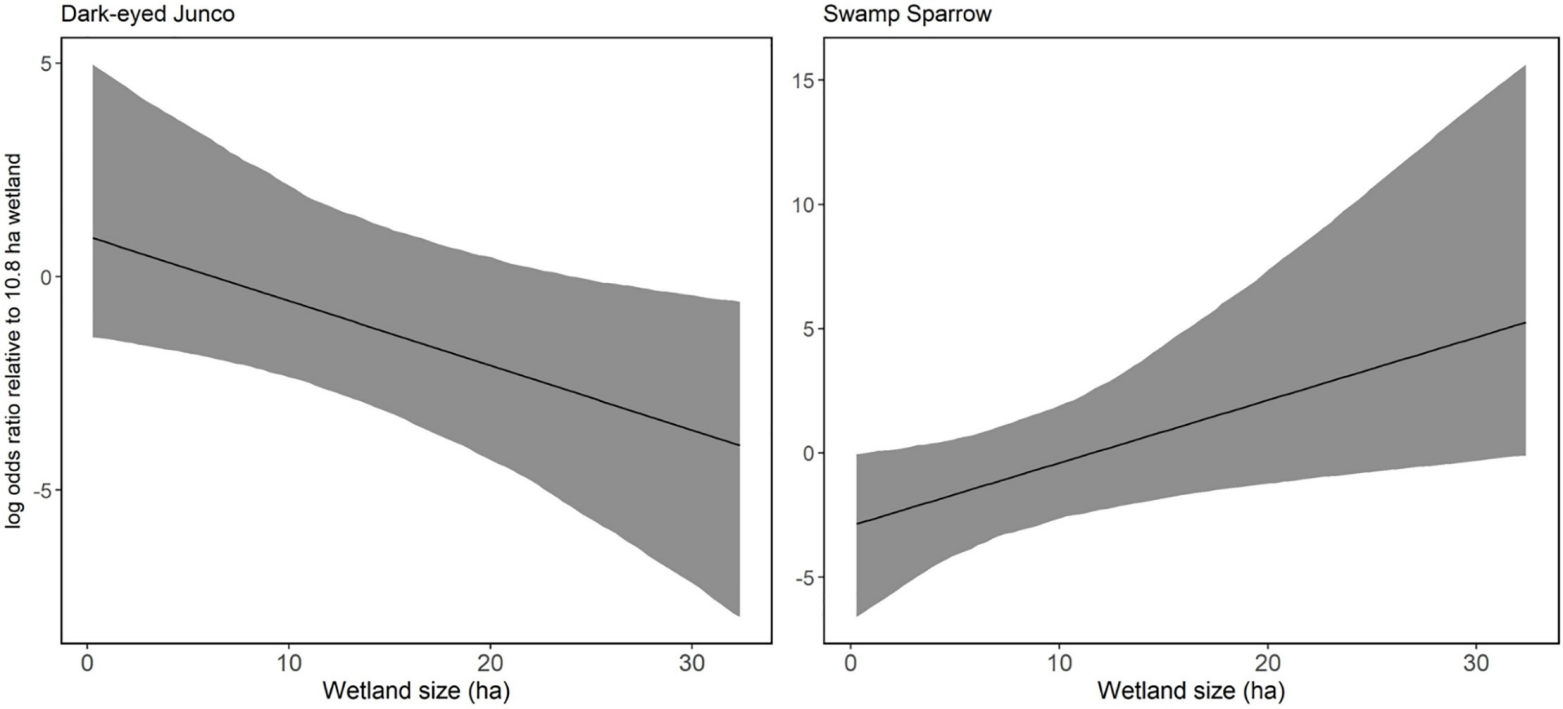
**Log odds ratio of dark-eyed junco occupancy(left) and swamp sparrow occupancy (right) as a function of wetland size.** Logs odds ratio is relative to median wetland size (10.8 ha) and gray bands represent 95% credible intervals from detection/non-detection surveys conducted in West Virginia, USA on Agricultural Conservation Easement Program wetlands and wetlands on public land in the winters of 2016–2017 and 2017–2018.

Despite failing to find differences in occupancy probability between ACEP and reference wetlands, we found that several environmental variables influenced site occupancy probabilities. For song sparrows, probability of occupying a site was greater when shrub coverage at the 1x1 m scale = 0–50% than when shrub coverage was >50–100% (log odds ratio = 2.6, 95% CI = 0.4–5.6). Additionally, song sparrow detection probability declined as winter progressed (log odds ratio = -1.1, 95% CI = -1.5—- 0.6). Dark-eyed junco occupancy was negatively associated with wetland size (log odds ratio = -1.6, 95% CI = -3.7—- 0.2) (Fig 4). Additionally, dark-eyed junco detection probability increased as the day progressed (log odds ratio = 0.8, 95% CI = 0.1–1.7). Swamp sparrow occupancy probability increased with wetland size (log odds ratio = 2.4, 95% CI = 0.5–6.1) (Fig 4), and detection probability was negatively associated with wind speed (log odds ratio = -0.9, 95% CI = -1.8—- 0.1). Finally, white-throated sparrow occupancy was positively associated with the presence of water (log odds ratio = 3.2, 95% CI = 0.2–7.2), but detection probability was not associated with any variables included in the model (i.e., 95% credible intervals of all detection variables overlapped 0). All other 95% credible intervals for the other slope coefficients included in all models for each species overlapped zero.

**Fig 5:**
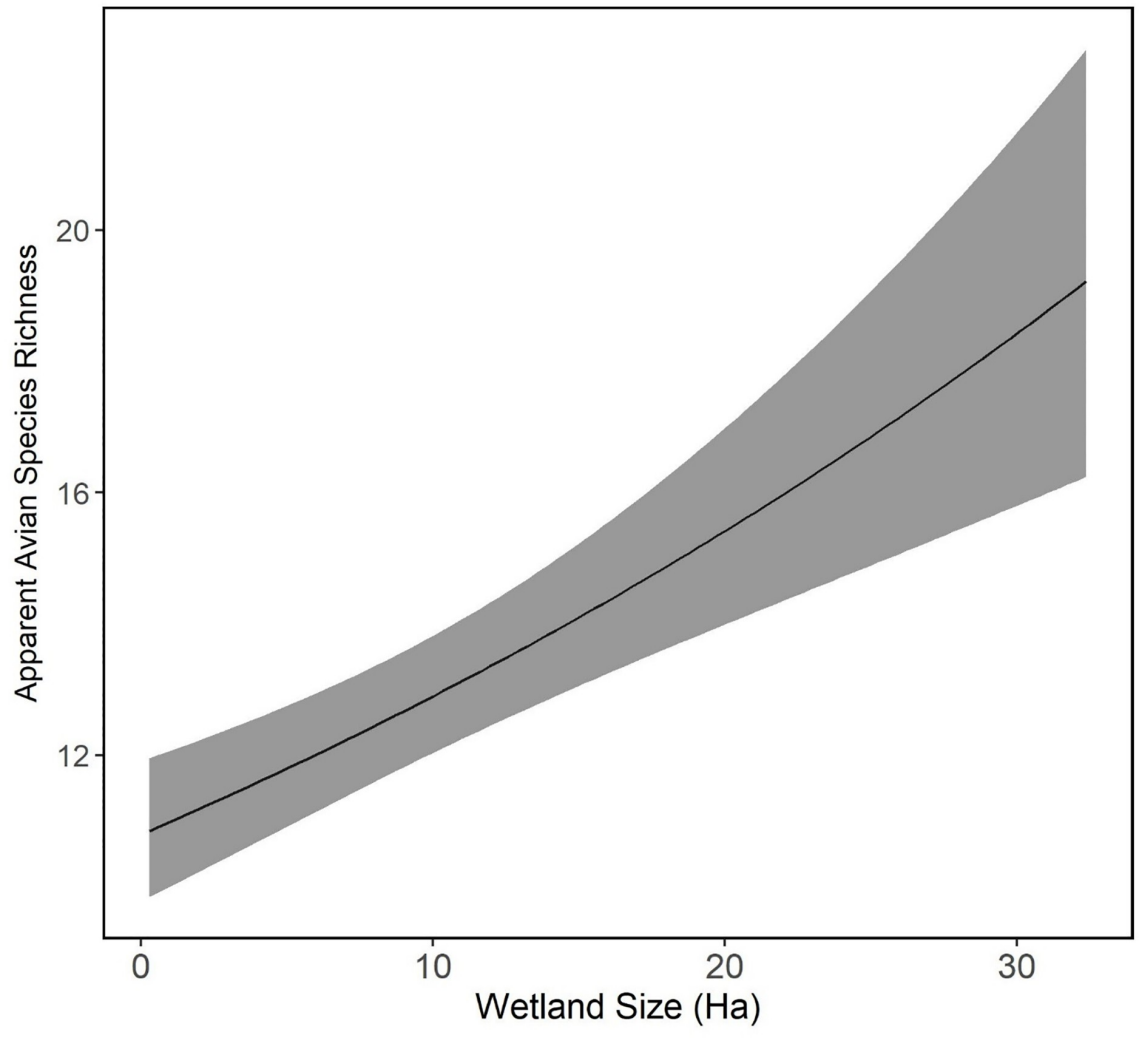
Apparent species richness probability on Agricultural Conservation Easement Program (ACEP) and reference wetland sites located in West Virginia, USA. Obtained from detection / non-detection survey data collected in the winters of 2016–2017 and 2017–2018. Apparent richness is modeled as a function of the top predictor we modeled: wetland size. Apparent species richness was positively correlated with wetland size.

We found no statistical difference in richness between years (log proportional change = -0.08, p-value > 0.10), or between ACEP and reference sites (log proportional change = -0.05, p-value > 0.10). Of the 61 total species we detected, 13 species were detected only at the ACEP wetland sites including American tree sparrow, cedar waxwing (*Bombycilla cedrorum*), Cooper's hawk (*Accipiter cooperii*), fox sparrow, green-winged teal (*Anas carolinensis*), house sparrow (*Passer domesticus*), yellow-rumped warbler (*Setophaga coronata*), osprey (*Pandion haliaetus*), ruby-crowned kinglet (*Regulus calendula*), Savannah sparrow, white-crowned sparrow, Wilson’s snipe (*Gallinago delicata*), and yellow-bellied sapsucker (*Sphyrapicus varius*) (S2 Table). Three species: winter wren (*Troglodytes hiemalis*), black vulture (*Coragyps atratus*), and mourning dove (*Zenaida macroura*) were only detected on reference sites (S2 Table). Apparent species richness was positively associated with wetland size (log proportional change = 0.2, p-value <0.01), (Fig 5).

## Discussion

We found that, after controlling for environmental variables, Passerellidae sparrows have similar site occupancy probabilities between ACEP wetlands on private agricultural property and reference wetlands on public lands. Further, we found no differences in overall species richness between ACEP and reference wetlands. This suggests that ACEP wetlands functionally provide similar habitat for wintering avifauna as publicly owned wetlands in West Virginia, even though ACEP wetlands were located primarily in open, agricultural areas and reference wetlands were located largely in forested areas. This indicates that ACEP wetlands in West Virginia are meeting program objectives and acting as an additional source of valuable wildlife habitat. In West Virginia, ACEP is the only conservation program that targets private, agricultural land and therefore provides a unique mechanism for restoring wetlands back on the landscape. Our findings are consistent with other studies comparing wildlife use of created or restored wetlands to naturally occurring wetlands [10–12]. Balcombe et al. [12] found avian abundance and richness were similar between mitigated and reference sites in the spring in West Virginia. Similarly, breeding bird abundance did not differ between restored and naturally occurring wetlands in New York, USA [43].

We found that wetland size had the strongest influence in wintering bird communities, as this covariate influenced occupancy probability of 2 sparrows and was positively associated with species richness. Our finding of greater swamp sparrow occupancy in larger wetlands is consistent with previous studies [15,22]. Swamp sparrows are more strongly associated with wetlands than the other Passerellidae species investigated here [44]. In the breeding season, swamp sparrows feed on aquatic invertebrates more so than any other Passerellidae species and build their nests in vegetation over water [45,46]. This association with wetlands then extends into the winter season. In contrast, we found a negative association between dark-eyed junco occupancy and wetland size. Dark-eyed juncos are a forest-associated species, breeding in coniferous stands and wintering along woodland edges [47]. Because dark-eyed juncos do not rely specifically on wetland habitat for the winter, a small amount of wetland habitat combined with other habitat types such as forested areas and open fields could fulfill multiple foraging, thermal cover, and sheltering habitat requirements. The ACEP and reference sites included in this study were most frequently surrounded by pasture and forest, respectively [8]. Smaller wetland sites may have been more appealing to wintering dark-eyed juncos because of the domination of pasture or forest in the surrounding area, as opposed to predominately wetland habitat in the area. Species that are not interior habitat specialists such as these sparrows may have higher densities in areas with increased edge habitat [48]. The ACEP sites in particular were frequently surrounded by pasture or agricultural fields, allowing for the potential for more edge habitat areas around the wetlands [8]. Alternatively, Schlossberg and King [49] found that dark-eyed juncos, along with other early successional bird species’ abundance was negatively associated with smaller patch sizes and proportion of edge habitat. This, however was not in relation to wetland habitat. Dark-eyed junco presence on smaller wetland sites could also be attributed to vegetative or site characteristics that occupied a different scale than the variables we measured. The dark-eyed junco negative association with wetland size suggests any amount of conserved wetland habitat within an agricultural landscape provides beneficial habitat for avian species, regardless of patch size. This has important implications for ACEP, as it would be important to enroll both large and small wetland areas as both provide benefits to wildlife.

Our finding that apparent avian species richness was positively correlated with wetland size is supported by earlier research. Previous studies of marsh bird communities have found similar increases in species richness as wetland size increased [16,22,50]. The correlation we found between avian species richness and wetland size is more broadly consistent with predictions from the species-area relationship, which postulates that species richness increases with patch size [51]. Because many of the sites enrolled in ACEP were isolated wetland areas within an agricultural matrix, these habitats could have acted as wetland “islands”. Larger wetlands had more available habitat and thus could have supported more species. Many avian species and wintering sparrows specifically partition habitat space to optimize foraging and protection from predation by foraging in flocks or close to cover [52]. Therefore, larger wetland areas could provide more foraging space for flocks as well as more vegetative cover.

In the winter, song sparrows and other Passerellidae species forage on the ground with varying amounts of shrub and tree cover [47]. Our finding that song sparrow occupancy probability was positively associated with <50% shrub cover likely reflects selection for sites with open ground for foraging along with a small amount of shrub coverage for shelter. Species such as song sparrows and white-throated sparrows selected winter foraging plots with dense screening cover or foraged in areas closer to screening cover to reduce the risk of predation in other studies [19,21]. Even small areas of herbaceous buffers can promote avian presence through all seasons [46, 53–55]. Our lack of finding any significant association with vegetative cover for the other species included in this study besides song sparrows is contrary to what others have found in the past. Swamp sparrows are commonly associated with dense shrub or woody coverage in the winter [56], and dark-eyed junco habitat use increased with increasing understory vegetation height in an exurban environment in Colorado, USA [57]. Based on our findings, it is possible that larger-scale characteristics such as wetland patch size were more important for some species in this study. It is also possible that our study did not evaluate the vegetative characteristics at a broad enough scale at each site to capture additional associations. Previous studies that have identified associations between species included in our study and vegetative characteristics evaluated understory cover in a 25 m radius [57], which covered a wider, less linear area than our vegetative measurements. Additionally, a study pertaining to swamp sparrow use of shrubs in the winter details seeking out shrub patches to survey swamp sparrows in order to increase the likelihood of detecting them [56]. These studies were focused on evaluating the habitat relationships of the sparrows, therefore they studied a broader range of variation in habitat variables. The primary focus of our study was a contrast between ACEP and reference wetlands, and we sought to statistically control for variation in other variables such as vegetative characteristics. Because of this, our study did not evaluate the same range of variation, which may explain our contrary results to previous studies.

The association we found between white-throated sparrow occupancy and the presence of surface water in wetland habitats has not been documented before. Within our study, the presence of standing water in the 1x1 m quadrats typically indicated a seasonal to semi-permanent hydroperiod. We did not observe white-throated sparrows in the water, rather we observed them using vegetation adjacent to areas that had a small amount of standing water.

White-throated sparrows are typically found in upland habitat and are not commonly associated with water or wetlands throughout the year [58,59]. Particularly in the winter, white-throated sparrows are habitat generalists and have been associated with both early successional, open habitat and denser shrubby areas [58]. Studies that have occurred during the breeding season have found similar indications that the white-throated sparrow is a generalist and can have varying responses to habitat treatments. Schlossberg et al. [59] found that white-throated sparrow abundance was negatively associated with shrub, forb, and tree vegetative characteristics in rural areas in western Massachusetts, USA, while Rousseau et al. [60] had high white-throated sparrow occupancy in both mature forests and clear-cuts in their study in Quebec, Canada. These variable associations throughout the year indicate that the white-throated sparrow may not have easily defined selection criteria for winter habitat. While the presence of water is an important settlement cue for other Passerellidae species, namely the swamp sparrow [45], there is little evidence that white-throated sparrows select wintering habitat based on the presence of water. Instead, it is possible that the white-throated sparrows in our study selected winter habitat based on different vegetative characteristics that were adjacent to small areas of standing water on the wetland.

Less than 1% of West Virginia’s surface is covered by wetlands. However, these wetlands provide crucial ecosystem services and contribute significantly to the state’s wildlife habitat [61]. ACEP establishes wetlands within an agricultural matrix on private land, which provides early successional wetland habitat that is an important component of wintering sparrow habitat. Our findings indicate that these sites are being used as much as other available wetland habitat in the state by avifauna, which suggests that ACEP is meeting avian habitat objectives. Additionally, our differing findings pertaining to wetland size indicate that any amount of restored wetlands can provide habitat for species located within agricultural and forested areas. Therefore, a continuation and expansion of ACEP in West Virginia could be a valuable source of wetland habitat. More broadly, this suggests set-aside conservation practices in agricultural landscapes may be an efficient method for conserving wetland habitat.

## Supporting information

S1 Table Agricultural Conservation Easement Program (ACEP) wetlands and reference wetlands located in West Virginia, USA. Table includes description of wetland size, county, physiographic region, wetland class, and number of point counts of each site. For ACEP sites, year of restoration is also included.
(PDF)

S2 Table Number of species detected standardized by number of point counts on Agricultural Conservation Easement Program (ACEP) and reference sites respectively to obtain percent of species ACEP and reference wetland sites located in West Virginia, USA obtained from detection / non-detection survey data collected in the winters of 2016–2017 and 2017–2018. (PDF)

S1 Dataset Detection and non-detection data used in occupancy analysis. Two replicate surveys of detection / non-detection data used in analysis for song sparrows (sosp), swamp sparrows (swsp), dark-eyed juncos (deju) and white-throated sparrows (wtsp) at each point count location during both survey years.
(CSV)

S2 Dataset Occupancy covariates used in occupancy analysis. Two replicate surveys of occupancy covariates collected at each point count location during both survey years. Occupancy covariates include wetland type (ACEP or reference), survey year (year), wetland size in ha (size), categorical herbaceous coverage at the 1x1 m scale (herb), categorical shrub coverage at the 1x1 m scale (shrub1), categorical bare-ground coverage at the 1x1 m scale (bareground), categorical woody vegetation coverage at the 1x1 m scale (woody), categorical water coverage at the 1x1 m scale (water), and categorical shrub coverage at the 5x5 m scale (shrub 5).
(CSV)

S3 Dataset Detection covariates used in occupancy analysis. Two replicate surveys of detection covariates collected during each survey occasion at each point count during both survey years. Detection covariates include time of day (time), sky condition (sky), wind speed (wind), temperature (temp), noise disturbance (dist), and day of year (day).
(CSV)

S4 Dataset Species richness counts used in apparent species richness analysis. Apparent species richness at each wetland site (richness), along with year surveyed (year), wetland type (type), and wetland size in ha (size).
(CSV)

S1 Analyses Passerellidae occupancy analysis and apparent species richness analysis. R file and containing code for all analyses done for the manuscript.
(R) Click here for additional data file.

S1 Models Model files for each species for occupancy analysis. PDF containing global and reduced models for each species used in JAGS occupancy analyses.
(PDF)

S1 Metadata Metadata file containing information for raw data supporting information files: S1 Dataset–S4 Dataset; S1 Analyses; S1 Models. (PDF)
