# Supplementary material for: Influence of the Agricultural Conservation Easement Program wetland practices on winter occupancy of Passerellidae sparrows and avian species richness
Source: PLoS One. 2019 Jan 24;14(1):e0210878. doi: 10.1371/journal.pone.0210878 (PMC6345491; doi:10.1371/journal.pone.0210878)
Supplement: S1 Table — Table includes description of wetland size, county, physiographic region, wetland class, and number of point counts of each site. For ACEP sites, year of restoration is also included. (PDF) [file pone.0210878.s001.pdf]

Site	Wetland Size (ha)	County	Physiographic Region	Wetland Class	Number of Point Counts	Year restored (ACEP)
**ACEP 1**	4.24	Summers	Appalachian Plateau	forested/scrub-shrub	2	2010
**ACEP 2**	1.32	Greenbriar	Appalachian Plateau	palustrine emergent	2	2010
**ACEP 3**	3.22	Greenbriar	Appalachian Plateau	forested / palustrine emergent	5	2012
**ACEP 4**	1.83	Upshur	Appalachian Plateau	palustrine emergent	4	1998
**ACEP 5**	3.88	Jefferson	Great Valley	forested/ palustrine emergent	3	1998
**ACEP 6**	18.6	Nicholas	Appalachian Plateau	scrub-shrub	1	1998
**ACEP 7**	6.58	Jefferson	Great Valley	forested/ palustrine emergent	4	1999
**ACEP 8**	6.11	Pendleton	Valley and Ridge	forested/ palustrine emergent	3	2011
**ACEP 9**	28.7	Mason	Appalachian Plateau	palustrine emergent	8	1996
**ACEP 10**	27.9	Nicholas	Appalachian Plateau	forested	5	1999
**ACEP 11**	0.405	Pendleton	Valley and Ridge	palustrine emergent	1	2010
**ACEP 12**	1.73	Jefferson	Great Valley	palustrine emergent	1	1998
**ACEP 13**	1.41	Clay	Appalachian Plateau	palustrine emergent	2	2001
**ACEP 14**	6.03	Pocahontas	Appalachian Plateau	scrub-shrub	5	1998
**ACEP 15**	0.911	Grant	Alleghany Mountain	palustrine emergent	3	2011
**ACEP 16**	16.8	Berkeley	Great Valley	palustrine emergent	7	1996
**ACEP 17**	3.68	Upshur	Appalachian Plateau	palustrine emergent	2	1999
**ACEP 18**	4.25	Preston	Alleghany Mountain	palustrine emergent	3	1998
**ACEP 19**	32.4	Barbour	Appalachian Plateau	palustrine emergent	9	1997

**ACEP 20**	3.83	Taylor	Appalachian Plateau	palustrine emergent	5	1998
**Reference 1**	4.54	Webster	Appalachian Plateau	forested/scrub shrub	3	
**Reference 2**	7.23	Monongalia	Appalachian Plateau	palustrine emergent	4	
**Reference 3**	25.2	Jefferson	Great Valley	palustrineemergent	7	
**Reference 4**	0.279	Webster	Appalachian Plateau	palustrine emergent	1	
**Reference 5**	9.71	Mason	Appalachian Plateau	palustrine emergent	4	
**Reference 6**	25.7	Greenbriar	Appalachian Plateau	forested /scrub-shrub	5	
**Reference 7**	3.44	Randolph	Alleghany Mountain	palustrineemergent	3	
**Reference 8**	6.70	Pocahontas	Appalachian Plateau	palustrine emergent	5	
**Reference 9**	14.3	Tucker	Alleghany Mountain	palustrine emergent/forested/scrub-shrub	6	
**Reference 10**	0.951	Upshur	Appalachian Plateau	palustrine emergent	1	
**Reference 11**	7.53	Preston	Alleghany Mountain	palustrine emergent	3	
**Reference 12**	2.91	Barbour	Appalachian Plateau	forested/scrub-shrub	2	
**Reference 13**	6.84	Barbour	Appalachian Plateau	forested/scrub -shrub	4	
