# Supplementary material for: Influence of the Agricultural Conservation Easement Program wetland practices on winter occupancy of Passerellidae sparrows and avian species richness
Source: PLoS One. 2019 Jan 24;14(1):e0210878. doi: 10.1371/journal.pone.0210878 (PMC6345491; doi:10.1371/journal.pone.0210878)
Supplement: S2 Table — (PDF) [file pone.0210878.s002.pdf]

Species	Percent detected on ACEP	Percent detected on Reference
American coot (*Fulica Americana*)	0.04	0
American crow (*Corvus brachyrhynchos*)	5.04	1.85
American goldfinch(*Spinus tristis*)	2.30	2.16
American robin (*Turdus migratorius*)	1.45	0.24
American tree sparrow (*Spizella arborea)*	0.05	0
Bald eagle *(Haliaeetus leucocephalus*)	0.03	0.03
Black-capped chickadee(*Poecile atricapillus*)	0.10	0.01
Belted kingfisher (*Megaceryle alcyon*)	0.13	0.15
Blue jay (*Cyanocitta cristata)*	2.10	0.95
Black vulture (*Coragyps atratus*)	0	0.03
Brown creeper (*Certhia americana*)	0.03	0.01
Carolina chickadee (*Poecile carolinensis*)	0	0.01
Canada goose (*Branta canadensis*)	0.58	1.10
Carolina wren (*Thryothorus ludovicianus*)	1.14	0.71
cedar waxwing (*Bombycilla cedrorum*)	0.27	0
Cooper's hawk (*Accipiter cooperii*)	0.01	0
Common raven (*Corvus corax*)	0.70	0.28
Dark-eyed junco (*Junco hyemalis*)	1.28	0.34
Downy woodpecker (*Picoides pubescens*)	0.35	0.49
Eastern bluebird *(Sialia sialis)*	1.34	0.47
Eastern phoebe (*Sayornis phoebe*)	0.04	0.01
Eastern towhee *(Pipilo erythrophthalmus*)	0.27	0.10
Tufted titmouse (*Baeolophus bicolor*)	0.30	0.33
European starling (*Sturnus vulgaris*)	3.33	0.04
Field sparrow (*Spizella pusilla*)	0.05	0.01
Fox sparrow (*Passerella iliaca*)	0.03	0
Great blue heron (*Ardea herodias*)	0.03	0.05
Golden crowned kinglet (*Regulus satrapa*)	0.10	0.11
Green-winged teal (*Anas carolinensis*)	0.05	0
Hairy woodpecker *(Leuconotopicus villosus*)	0.03	0.038
House finch *(Haemorhous mexicanus*)	0.04	0.01
House sparrow (*Passer domesticus*)	0.03	0
Killdeer (*Charadrius vociferus*)	0.05	0.01
Mallard (*Anas platyrhynchos*)	0.13	0.15
Mourning dove (*Zenaida macroura*)	0	0.09
Northern cardinal (*Cardinalis cardinalis*)	0.92	0.47
Northern flicker (*Colaptes auratus*)	0.16	0.05
Northern mockingbird (*Mimus polyglottos*)	0.30	0.05
Osprey (*Pandion haliaetus*)	0.04	0
Pied-billed grebe (*Podilymbus podiceps*)	0.01	0.01
Pine siskin (*Spinus pinus*)	0	0.03
Pileated woodpecker (*Hylatomus pileatus*)	0.34	0.13

Purple finch (*Haemorhous purpureus*)	0	0.03
Red-bellied woodpecker (*Melanerpes carolinus*)	0.14	0.15
Ruby-crowned kinglet (*Regulus calendula*)	0.01	0
Red shouldered hawk *(Buteo lineatus*)	0.19	0.04
Red tailed hawk (*Buteo jamaicensis*)	0.08	0.05
Ruffed grouse *(Bonasa umbellus*)	0.05	0.01
Red-winged blackbird (*Agelaius phoeniceus*)	0.08	0.10
Savannah sparrow (*Passerculus sandwichensis*)	0.01	0
Song sparrow (*Melospiza melodia*)	5.38	1.53
Sharp-shinned hawk (*Accipiter striatus*)	0.03	0.01
Swamp sparrow (*Melospiza georgiana*)	0.73	0.13
Turkey vulture *(Cathartes aura*)	0.54	1.24
Unknown chickadee (*Poecile spp.*)	2.29	1.78
White-breasted nuthatch (*Sitta carolinensis*)	0.81	0.67
White-crowned sparrow (*Zonotrichia leucophrys*)	0.06	0
Wilson's snipe (*Gallinago delicata*)	0.03	0
Winter wren (*Troglodytes hiemalis*)	0.01	0.03
Wood duck (*Aix sponsa*)	0.03	0.01
White-throated sparrow (*Zonotrichia albicollis*)	1.42	0.68
Yellow-bellied sapsucker (*Sphyrapicus varius*)	0.01	0
Yellow-rumped warbler (*Setophaga coronata*)	0.04	0
