# Supplementary material for: Influence of the Agricultural Conservation Easement Program wetland practices on winter occupancy of Passerellidae sparrows and avian species richness
Source: PLoS One. 2019 Jan 24;14(1):e0210878. doi: 10.1371/journal.pone.0210878 (PMC6345491; doi:10.1371/journal.pone.0210878)
Supplement: S1 Models — PDF containing global and reduced models for each species used in JAGS occupancy analyses. (PDF) [file pone.0210878.s008.pdf]

Song sparrow global model ("sosp.txt"):

```
model{
for(c in 1:12){
  b[c] ~ dlogis(0, 1)
}
for(d in 1:7){
  a[d] ~ dlogis(0, 1)
}
for(i in 1:N){
  z[i] ~ dbern(psi[i])
  logit(psi[i]) <- b[1] + b[2] * acep[i] + b[3] * yr[i] + b[4] * size[i] +
                b[5] * herb1[i] + b[6] * herb2[i] + b[7] * shrub[i] +
                b[8] * bg[i] + b[9] * wood[i] + b[10] * water[i] +
                b[11] * sh1[i] + b[12] * sh2[i]

  for(j in 1:J){
    y[i, j] ~ dbern(mu[i, j])
    mu[i, j] <- p[i, j] * z[i]
    logit(p[i, j]) <- a[1] + a[2] * time[i, j] + a[3] * sky[i, j] +
                 a[4] * wind[i, j] + a[5] * temp[i, j] +
                 a[6] * dist[i, j] + a[7] * day[i, j]

    }

  }

}
```

Song sparrow reduced model (“sosp2.txt”)

```
model{
 for(c in 1:6){
  b[c] ~ dlogis(0, 1)
 }
 for(d in 1:7){
  a[d] ~ dlogis(0, 1)
 }
 for(i in 1:N){
  z[i] ~ dbern(psi[i])
  logit(psi[i]) <- b[1] + b[2] * acep[i] + b[3] * size[i] + b[4] * shrub[i] +
                  b[5] * sh1[i] + b[6] * sh2[i]
  for(j in 1:J){
   y[i, j] ~ dbern(mu[i, j])
   mu[i, j] <- p[i, j] * z[i]
   logit(p[i, j]) <- a[1] + a[2] * time[i, j] + a[3] * sky[i, j] +
                    a[4] * wind[i, j] + a[5] * temp[i, j] +
                    a[6] * dist[i, j] + a[7] * day[i, j]

  }

 }

}
```

Dark-eyed junco global model (“deju.txt”):

```
model{
 for(c in 1:12){
  b[c] ~ dlogis(0, 1)
 }
 for(d in 1:7){
  a[d] ~ dlogis(0, 1)
 }
 for(i in 1:N){
  z[i] ~ dbern(psi[i])
  logit(psi[i]) <- b[1] + b[2] * acep[i] + b[3] * yr[i] + b[4] * size[i] +
                 b[5] * herb1[i] + b[6] * herb2[i] + b[7] * shrub[i] +
                 b[8] * bg[i] + b[9] * wood[i] + b[10] * water[i] +
                 b[11] * sh1[i] + b[12] * sh2[i]
  for(j in 1:J){
   y[i, j] ~ dbern(mu[i, j])
   mu[i, j] <- p[i, j] * z[i]
   logit(p[i, j]) <- a[1] + a[2] * time[i, j] + a[3] * sky[i, j] +
                  a[4] * wind[i, j] + a[5] * temp[i, j] +
                  a[6] * dist[i, j] + a[7] * day[i, j]


   }


  }


 }
```

Dark-eyed junco reduced model ("deju2.txt")

```
model{
  for(c in 1:4){
    b[c] ~ dlogis(0, 1)
  }
  for(d in 1:6){
    a[d] ~ dlogis(0, 1)
  }
  for(i in 1:N){
    z[i] ~ dbern(psi[i])
    logit(psi[i]) <- b[1] + b[2] * size[i] +
                  b[3] * sh1[i] + b[4] * sh2[i]
    for(j in 1:J){
      y[i, j] ~ dbern(mu[i, j])
      mu[i, j] <- p[i, j] * z[i]
      logit(p[i, j]) <- a[1] + a[2] * time[i, j] + a[3] * sky[i, j] +
                  a[4] * wind[i, j] + a[5] * temp[i, j] +
                  a[6] * dist[i, j]

      }

    }

  }
```

Swamp sparrow global model (“swsp.txt”)

```
model{
  for(c in 1:12){
    b[c] ~ dlogis(0, 1)
  }
  for(d in 1:7){
    a[d] ~ dlogis(0, 1)
  }
  for(i in 1:N){
    z[i] ~ dbern(psi[i])
    logit(psi[i]) <- b[1] + b[2] * acep[i] + b[3] * yr[i] + b[4] * size[i] +
                  b[5] * herb1[i] + b[6] * herb2[i] + b[7] * shrub[i] +
                  b[8] * bg[i] + b[9] * wood[i] + b[10] * water[i] +
                  b[11] * sh1[i] + b[12] * sh2[i]

    for(j in 1:J){

      y[i, j] ~ dbern(mu[i, j])
      mu[i, j] <- p[i, j] * z[i]
      logit(p[i, j]) <- a[1] + a[2] * time[i, j] + a[3] * sky[i, j] +
                    a[4] * wind[i, j] + a[5] * temp[i, j] +
                    a[6] * dist[i, j] + a[7] * day[i, j]

    }

  }

}
```

Swamp sparrow reduced model ("swsp2.txt")

```
model{
  for(c in 1:7){
    b[c] ~ dlogis(0, 1)
  }
  for(d in 1:4){
    a[d] ~ dlogis(0, 1)
  }
  for(i in 1:N){
    z[i] ~ dbern(psi[i])
    logit(psi[i]) <- b[1] + b[2] * yr[i] + b[3] * size[i] +
                     b[4] * herb1[i] + b[5] * herb2[i] + b[6] * shrub[i] +
                     b[7] * water[i]

    for(j in 1:J){

      y[i, j] ~ dbern(mu[i, j])
      mu[i, j] <- p[i, j] * z[i]
      logit(p[i, j]) <- a[1] +
                        a[2] * wind[i, j] +
                        a[3] * dist[i, j] + a[4] * day[i, j]

    }

  }

  }
```

White-throated sparrow global model (“wtsp.txt”):

```
model{
  for(c in 1:12){
    b[c] ~ dlogis(0, 1)
  }
  for(d in 1:7){
    a[d] ~ dlogis(0, 1)
  }
  for(i in 1:N){
    z[i] ~ dbern(psi[i])
    logit(psi[i]) <- b[1] + b[2] * acep[i] + b[3] * yr[i] + b[4] * size[i] +
                 b[5] * herb1[i] + b[6] * herb2[i] + b[7] * shrub[i] +
                 b[8] * bg[i] + b[9] * wood[i] + b[10] * water[i] +
                 b[11] * sh1[i] + b[12] * sh2[i]

    for(j in 1:J){

      y[i, j] ~ dbern(mu[i, j])
      mu[i, j] <- p[i, j] * z[i]
      logit(p[i, j]) <- a[1] + a[2] * time[i, j] + a[3] * sky[i, j] +
                  a[4] * wind[i, j] + a[5] * temp[i, j] +
                  a[6] * dist[i, j] + a[7] * day[i, j]

    }

  }

}
```

White-throated sparrow reduced model (“wtsp2.txt”)

```
model{
  for(c in 1:5){
   b[c] ~ dlogis(0, 1)
  }
  for(d in 1:4){
   a[d] ~ dlogis(0, 1)
  }
  for(i in 1:N){

   z[i] ~ dbern(psi[i])
   logit(psi[i]) <- b[1] + b[2]  * size[i] +
                b[3] * herb1[i] + b[4] * herb2[i] +  b[5] * water[i]
   for(j in 1:J){

     y[i, j] ~ dbern(mu[i, j])
     mu[i, j] <- p[i, j] * z[i]
     logit(p[i, j]) <- a[1] + a[2] * temp[i, j] +
                  a[3] * dist[i, j] + a[4] * day[i, j]

     }

   }

  }
```
