# Supplementary material for: Influence of the Agricultural Conservation Easement Program wetland practices on winter occupancy of Passerellidae sparrows and avian species richness
Source: PLoS One. 2019 Jan 24;14(1):e0210878. doi: 10.1371/journal.pone.0210878 (PMC6345491; doi:10.1371/journal.pone.0210878)
Supplement: S1 Metadata — (PDF) [file pone.0210878.s009.pdf]

Metadata:

S1 Dataset:

point: point count location

sosp.1, swsp.1, deju.1, wtsp.1: detection/non-detection data during the first of 2 rounds of surveys per survey year for song sparrows, swamp sparrows, dark-eyed juncos, and white-throated sparrows

sosp.2, swsp.2, deju.2, wtsp.2: detection/non-detection data during the second of 2 rounds of surveys per survey year for song sparrows, swamp sparrows, dark-eyed juncos, and white-throated sparrows

S2 Dataset:

point: point count location

type: categorical: wetland type, acep or reference

year: categorical: 0 denotes year 1, 1 denotes year 2

size: continuous: wetland size in ha

herb: categorical: herbaceous coverage at the 1x1 m scale at each point count location

shrub1: categorical: shrub coverage at the 1x1 m scale at each point count location

bareground: categorical: bare-ground coverage at the 1x1 m scale at each point count location

woody: categorical: woody vegetation coverage at the 1x1 m scale at each point count location

water: categorical: water coverage at the 1x1 m scale at each point count location

shrub5: categorical: shrub coverage at the 5x5 m scale at each point count location

S3 Dataset:

point: point count location

time.1: Continuous: time of day that the point count started during the first of 2 rounds of replicate surveys

time.2: Continuous: time of day that the point count started during the second of 2 rounds of replicate surveys

sky.1: Categorical: sky condition during the first of 2 rounds of replicate surveys

sky.2: Categorical: sky condition during the second of 2 rounds of replicate surveys

wind.1: Continuous: wind speed in m/s during the first of 2 rounds of replicate surveys

wind.2: Continuous: wind speed in m/s during the second of 2 rounds of replicate surveys

temp.1: Continuous: temperature in Celsius during the first of 2 rounds of replicate surveys

temp.2: Continuous: temperature in Celsius during the second of 2 rounds of replicate surveys

dist.1: Continuous: noise disturbance measured in decibels during the first of 2 rounds of replicate surveys

dist.2: Continuous: noise disturbance measured in decibels during the second of 2 rounds of replicate surveys

day.1: Continuous: day of year that the first of 2 rounds of replicate point count surveys occurred on

day.2: Continuous: day of year that the second of 2 rounds of replicate point count surveys occurred on

S4 Dataset:

site: ACEP or reference site where richness was recorded

year: first year of surveys (1) and second year of surveys (2)

type: acep or reference

size: Continuous: wetland size in ha

richness: count of species detected at each site

S1 Analyses:

File contains all code for all analyses conducted starting with occupancy analyses of song sparrows, darkeyed juncos, swamp sparrows, and white-throated sparrows. Also includes analysis conducted for apparent species richness. S3-S6 datasets correspond to analyses

S1 Models:

Global (containing all covariates) and reduced (containing those covariates whose 50% credible intervals do not cross zero) models for each of the Passerellidae species included in this study used for JAGS occupancy analysis.
